# Experiences of Everyday Life among Individuals with Co-Existence of Serious Mental Illness and Cancer—A Qualitative Systematic Literature Review

**DOI:** 10.3390/healthcare11131897

**Published:** 2023-06-30

**Authors:** Stinne Glasdam, Lisbeth Hybholt, Sigrid Stjernswärd

**Affiliations:** 1 Department of Health Sciences, Faculty of Medicine, Lund University, 222 41 Lund, Sweden; stinne.glasdam@med.lu.se; 2 Research Unit, Mental Health Services East, Psychiatry Region Zealand, Smedegade 16, 4000 Roskilde, Denmark; lihy@regionsjaelland.dk; 3 Psychiatric Research Unit, Psychiatry Region Zealand, Fælledvej 6, 4200 Slagelse, Denmark

**Keywords:** cancer, comorbidity, encounters, everyday life, healthcare, severe mental illness, qualitative systematic literature review

## Abstract

Daily life with severe mental health (SMI) and cancer comorbidity entails multiple challenges. The study aims to explore everyday life experiences among individuals with SMI and cancer comorbidity from the perspectives of patients, significant others, and involved healthcare professionals. The study is registered in PROSPERO (CRD42021259604). A qualitative systematic review was conducted through searches in the databases MEDLINE, CINAHL, PsychInfo, and Web of Sciences (last search 14 February 2023). Inclusion criteria were empirical qualitative research studies investigating experiences of healthcare and everyday life among persons living with SMI and who were subsequently diagnosed with cancer from the perspective of the individuals themselves, their significant others, and healthcare professionals involved in their care. Exclusion criteria: Literature reviews, quantitative studies, intervention studies, quantitative parts of mix-methods studies, non-English languages, persons <18 years, dementia/learning disabilities, diagnosed with anxiety/depression as a consequence of cancer. Seven articles, published between January 2011 and February 2023, were included and analysed through a thematic analysis. The PRISMA 2020 checklist guided the study. The results were presented in four themes: ‘Navigating between different worlds and logics’, ‘Decision-making capacity depending on the assessor’, ‘Cancer must give way to severe mental illness or vice versa’, and ‘Significant others as a safety net’. Research about the everyday lives of persons with SMI and cancer comorbidities from patients’ and relatives’ perspectives is lacking and thus called for.

## 1. Introduction

Individuals with severe mental illness (SMI) have a two to three times higher mortality rate, reduced life expectancy, and poorer physical health compared to the general population [1,2,3]. In the current article, SMI is defined in line with Millman et al. [4] as “schizophrenia, other non-organic psychoses and major affective disorders including bipolar disorder and major depressive disorder”. Individuals with SMI show a higher risk of being diagnosed with cancer at advanced stages, including a delay in cancer diagnosis [5,6,7] and lower rates of cancer screening compared to the general population [8,9], which can be related to barriers such as information, poor staff–client relationships, travel difficulties, etc. [8,10]. Leahy et al.’s [11] review shows that uncontrolled psychiatric symptoms and their adverse impact on engaging with cancer care, and also stigmatising attitudes from staff and systems barrier-fragmentation, were barriers to cancer screening and treatment in individuals with concurrent SMI and cancer. Opposite, controlled psychiatric symptoms and existent connections with mental health services could facilitate such care, as could an enhanced collaboration between healthcare professionals across care sectors and the development of a patient navigator role embraced by supportive staff members [11]. A study of comorbidity in patients with schizophrenia and cancer also shows that comorbidity can both complicate and improve coping with both conditions, with the authors arguing for the need for additional studies to develop and customise effective psychosocial interventions for individuals with comorbidity [12]. Once diagnosed with cancer, individuals with SMI are more likely to die of cancer than others [1,5,13]. However, a study shows that mental disorders do not affect the prognosis of gynaecological cancers, except for advanced cervical cancer [14]. Cancer treatment plans have also been shown to differ, with more invasive interventions and lesser frequencies of chemotherapy or advanced diagnostic examinations in persons with SMI and cancer compared to others, indicating the presence of healthcare disparities and influencing end-of-life care quality in these patients [15].

SMI can negatively affect individuals’ cognitive abilities including memory, volition, and ability to articulate needs in daily life [16,17], which can impede help-seeking, disease insight, and adherence to treatment. High rates of smoking, alcohol consumption, and psychotropic drugs-related effects with sedation, sedentary lifestyles, and obesity in persons with SMI can affect health negatively [10,18,19]. Adherence to pharmacological treatment can be affected by disease symptoms, previous experience with specific drugs, and debilitating secondary effects of treatment [20,21]. Yet, the main reason for early death among individuals with SMI is somatic disease [22]. In several countries, psychiatric and somatic care are organisationally separated. Additionally, the responsibility for some functions can be located at the national/regional levels and for other functions at the municipal/local levels [23], leading to a shared responsibility for psychiatric care and support between county councils that provide healthcare services and municipalities that provide social care services [24]. This shared responsibility comes with an embedded risk that individuals with SMI and somatic comorbidities fall between the cracks, where no professionals take responsibility for complex comorbidity problems [23]. Individuals with SMI and cancer experience challenges complying with treatment and a disconnection and lack of collaboration between services [25,26], such as psychiatric care and medicine, and primary and specialised care [27].

Physical illness in individuals with SMI is often underdiagnosed, with physical symptoms being interpreted as pharmacological side effects or psychiatric symptoms [28]. In addition, experiences of (self) stigma and discrimination can hinder help-seeking in persons with SMI [27,28], which may also prevent healthcare professionals from properly addressing somatic health conditions in these patients. Varying living conditions in individuals with SMI, such as supported housing facilities or homelessness, can affect their access to social support and healthcare in daily life [29].

The area of SMI comorbidities most studied in cancer patients is unipolar depression, which is associated with difficulties with participating in and benefitting from cancer treatments [30,31]. Moreover, individuals with SMI are often excluded from cancer research, e.g., [32,33], and they rarely make themselves heard in health research in general [1]. Millman and colleagues [4] argue that there is insufficient research addressing the experiences of cancer and cancer services from the perspective of patients with SMI, which requires attention from policymakers, healthcare professionals, and researchers to address an important transdisciplinary gap in healthcare research [34]. To our knowledge, only one literature review has been undertaken in this area, which identifies key barriers and facilitators to accessing cancer care for individuals with cancer and SMI comorbidities [11]. An understanding of the everyday life of patients with SMI and cancer comorbidity is necessary to understand and support these patients and to effectively address their challenges in the healthcare system, motivating the current systematic review of the newest literature on the subject. This review focuses on individuals with both SMI and cancer diagnosis when they are enrolled in cancer diagnostics, treatment, and follow-up and/or cancer care. With an interest in understanding the complexity of everyday life marked by simultaneous severe somatic conditions and SMI, this review aims to explore everyday life experiences among individuals with the co-existence of SMI and cancer from the perspective of patients, their significant others, and healthcare professionals involved in their care.

## 2. Methods

This study was based on a qualitative systematic literature review synthesising findings across qualitative studies [35] through a Braun and Clarke [36] inspired thematic analysis. The review is registered in PROSPERO (CRD42021259604). The study is guided by the PRISMA checklist [37].

### 2.1. Review Questions

The focused review question was: From the perspectives of patients, significant others, and healthcare professionals, how were everyday life experiences among individuals with the co-existence of SMI and cancer expressed?

### 2.2. Search Strategy

A search was conducted with the search engine PubMed by primarily searching the MEDLINE database, and the databases CINAHL and PsycINFO, with guidance from two experienced librarians. A building block search was used based on the population, exposure, and outcome (PEO) model [35] (Table 1). Each block included search terms customised to each database. The search was limited to research articles written in English and published from 1 January 2011 to 14 February 2023. The full electronic search string is available in Appendix A. To find relevant studies not found through the current search strings in the listed databases, a citation pearl search was conducted in the Web of Science citation database to identify more relevant publications for the current literature review. The included articles’ references were used in two ways in the Web of Science: (1) to check if the included articles’ references also were relevant publications to include in the current review and (2) to find newer publications citing the included articles, and assessing if these newer publications were relevant to include in the current review.

### 2.3. Inclusion and Exclusion Criteria

**Figure 1:**
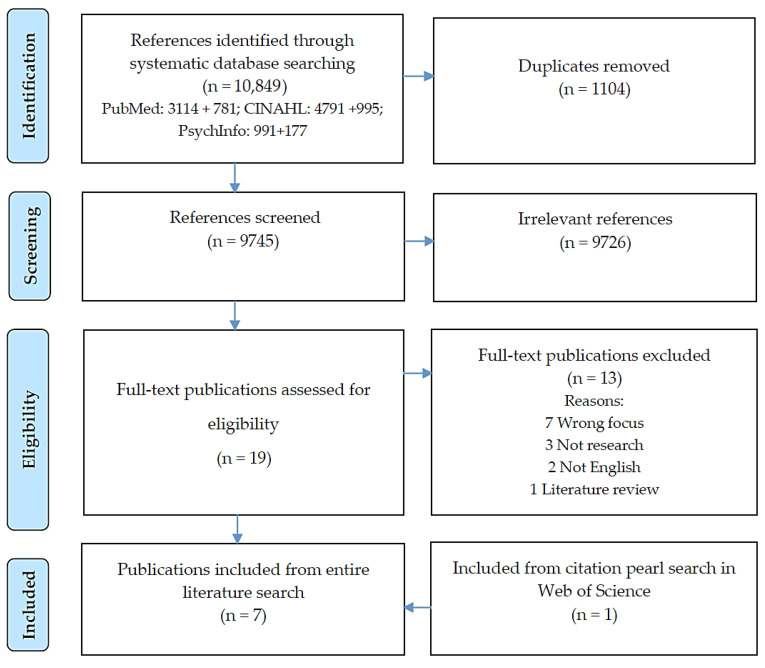
Flow chart illustrating the literature search process.

Two authors (SG, LH) separately screened 10,489 articles, titles, abstracts, and full texts using COVIDENCE.org. The inclusion criteria were: empirical qualitative research studies investigating experiences of healthcare and everyday life among persons living with SMI and who were subsequently diagnosed with cancer from the perspective of the individuals themselves, their significant others, and healthcare professionals involved in their care. Exclusion criteria were: literature reviews, quantitative studies, intervention studies, quantitative parts of mix-methods studies, articles in non-English languages, persons < 18 years old (patients/relatives), persons with dementia/learning disabilities, and persons who were diagnosed with anxiety/depression as a consequence of cancer. The two authors discussed the decision to include/exclude articles with the third author (SS) until agreement was reached whenever there were discrepancies in the screening process. The review included six articles. After the citation pearl search in Web of Science, one supplementary article was included, see Figure 1. The seven included studies are marked with an asterisk* in the references.

### 2.4. Selecting, Appraising, and Extracting Relevant Data

The first and last authors (SG, SS) performed the data extraction and coding stages. Based on the Cochrane Consumers and Communication Review Groups’ data extraction template [38], a structured data extraction spreadsheet was created including: (1) Authors, profession (2) Year, (3) Country, (4) Journal, (5) Impact factor (extracted from journal website), (6) Study period, (7) Study design, (8) Sample size, (9) Study population, (10) Theory and concepts, and (11) Results. Table 2 shows a selection of the data. Extracted data were validated through an iterative process in which comparisons were made repeatedly between the extracted data and the original articles. In case of discrepancies or if central nuances were missing in the table, the authors further discussed the data. For quality assessment of the studies, the authors used the Joanna Briggs Institute’s [39] (JBI) Checklist for Case Reports and Checklist for Qualitative Research.

### 2.5. Strategy for Synthesising Data

The analytical synthesis of data across the included studies consisted of a descriptive numerical summary analysis presented as ‘*Study Characteristics*’ and a Braun and Clarke [36] inspired inductive, thematic analysis where the included studies’ results are integrated and synthesised through a process including five phases: familiarisation with the data, generation of initial codes, search for themes, review of themes, and definition and naming of themes. First, the articles were read multiple times for familiarisation with the included material. Second, the articles were coded and reorganised in accordance with the current review’s research questions and aims. We chose to consider both the case descriptions and associated discussions/commentaries as empirical material in the case studies, as both were constructed from the authors’/professionals’ perspectives and the cases served to support the discussions. In the interview study, only the results section was included as empirical material for analysis. We treated the selected data similarly, in the sense that all included material as described here was treated as empirical material. Third, the initial themes were constructed from the coded material, based on similarities and differences in the coded material. Similar codes were constructed into themes, thereby synthesising data across the included studies. Fourth, the themes were reviewed and further developed through a mutual and consensual process amongst the authors, ensuring that the themes appropriately reflected the empirical material in its totality and that the analysis reflected the current article’s aim. In line with Bettany-Saltikov and McSherry [35], the included studies were described, integrated, and synthesised through interpretations of the primary studies to generate new interpretive understandings of everyday life experiences among individuals with co-existence of SMI and cancer. The analysis process thus encompassed a movement towards higher abstraction levels and synthesis, including the authors’ interpretations of data across the included studies. Finally, the four final themes were refined, defined, and named: ‘*Navigating between different worlds and logics*’, ‘*Decision-making capacity depending on the assessor’*, ‘*Cancer must give way to severe mental illness or vice versa*’, and ‘*Significant others as a safety net*’.

## 3. Findings

### 3.1. Study Characteristics

Three of the studies were carried out between 2009 and 2019. The study periods of the remaining studies were not reported. The studies were conducted in the USA (*n* = 5), South Africa (*n* = 1), and New Zealand (*n* = 1). Two articles were published in oncological journals, the others in journals of medicine, family practices, ethics, surgery, and social sciences, respectively. The journals’ impact factors (IF) ranged from 0.98 to 1.576 (two journals had no IF reported). Five studies were conducted by physicians from different specialisations [40,41,42,43,45], one by a public health physician and a researcher in applied public health [44], and one by oncological and psychiatric nurses [37]. For a schematic overview, please see Table 2.

Six studies were descriptive case studies from the perspectives of physicians and nurses [40,41,42,43,45,46]. One study was a semi-structured interview study investigating the perspectives of patients [44]. No studies from the perspectives of significant others were found. Altogether, ten patients were interviewed, and eleven patient cases were described.

Six of the studies highlighted the challenges experienced by healthcare professionals in their encounters with individuals with SMI and cancer comorbidities, predominantly from a cancer care perspective. Several issues were highlighted, including communication challenges and views on the patients’ (in)abilities to understand their situations and make health-related decisions [40,41,42,43], aspects relating to adherence to treatment and potential complications related to comorbidity [41,44], ethical challenges [42,43,45], and challenges allotted to the patients’ psychiatric condition, affecting health related values, consent, and adherence to treatment [41,42,45]. The studies also highlighted issues pertaining to medical complexities, psychopharmacological interactions, and effects on symptoms [41,43,46].

Four of the case studies followed the applicable criteria in the JBI critical appraisal checklist for case reports. Two case studies [40,45] lacked a clear description of the patients’ histories over time. All case studies provided takeaway lessons related to the current review’s research question. The qualitative interview study [44] followed the JBI criteria for qualitative research except that it lacked statements locating the researchers culturally and did not address the researchers’ influence on the research process.

### 3.2. Navigating between Different Worlds and Logics

The starting point in all studies was that the patient already had an SMI and from a somatic perspective, it was necessary to focus on the cancer disease. This raised several challenges for both patients and professionals. Guan et al. [41] showed how multidisciplinary collaboration between surgeons, a psychiatrist, and nursing home staff benefited a patient with schizophrenia and colon-rectal cancer, as seen from the physicians’ perspective. Contrarily, the disconnection between cancer and mental health services in New Zealand was problematised, describing the two services as separate [44]. While this felt problematic for some patients, it did not bother others. At times, it was experienced as an advantage, leading to personal control over the information flow, with patients acting as a link between the respective services [44]. Some patients reported positive experiences in connection with cancer care and contrasted these with negative experiences from mental health services. Clinicians in the cancer care services took the patients’ mental health concerns seriously, without stigmatisation from the patients’ perspective [44]. Other patients used their general practitioner (GP) as a bridge builder between cancer care and psychiatric services to support them. The patients and the physicians faced difficulties in managing multiple conditions and medications, as both SMI and cancer caused multiple problems to manage in daily life [44,46].

Differing views and priorities between different physicians, patients, and significant others regarding health and treatment could create tensions when opting for or against cancer treatment [42,43]. This could lead to opposite treatment decisions, e.g., opting for or against surgical interventions. Furthermore, the lack of trust in the patients’ abilities to make informed decisions and adhere to treatment came through as arguments to deny cancer treatment. Cole and Padmanabhan [40] focussed on three patients in their study. One patient was described as having suboptimal treatment of his/her psychiatric disease, refusing adjuvant chemotherapy and radiation, showing poor adherence to adjuvant hormonal therapy, and an inability to keep scheduled visits. Another patient was described as refusing staging scans, intravenous chemotherapy, or surgery, and as insisting that clinical appointments did not exceed one visit/month. The third patient was characterised as having suboptimal control of his/her psychiatric disease and not adhering to cancer treatments [40]. Taylor and colleagues [45] expressed that psychiatric care was not included within cancer care services, as cancer care professionals could not cater to patients’ psychiatric care needs. Furthermore, patients experienced that cancer care services were not aware of their mental health history [44]. Stabilisation of the SMI through psychiatric services was, however, deemed a priority before further oncological treatment to achieve successful outcomes [40,41], which sometimes required involuntary psychiatric treatment [45]. Thomson and Henry [46] showed how cancer care services tried to incorporate psychiatric care in their practice, where a woman with schizophrenia got extended appointments to lessen psychological discomfort and to support a structured treatment. However, the patient withdrew from cancer treatment after 12 weeks [46]. In their case, Guan et al. [41] also gave a practical example of how psychiatric care could be included in oncological care.

Healthcare professionals reported frustration and difficulties communicating with patients. They allotted such difficulties to the patients’ psychiatric conditions with associated symptoms and subsequent effects on cognitive abilities (e.g., self-neglect, deviant behaviour, lack of trust), treatment adherence, and disease insight [40,41,43]. Six out of seven studies showed that some patients were met with distancing, non-understanding, and anxiety from healthcare professionals. Professionals in cancer care labelled them as ‘non-compliant’ patients [40,42,43,44,45,46].

Still, some professionals within cancer care services were prompt to act upon existential challenges such as loneliness. There was a tendency to act on the need to ‘fix’ the patient or offer an immediate, practical solution. The patients were met with a professional mindset encouraging activities and self-responsibility. Thomson and Henry [46] showed how a woman with major depression and cancer was guided to participate in a professionally initiated cancer wellness program because of her feelings of loneliness. This patient had problems with excessive drinking and disheartening romances. She had lost contact with friends when she completed her cancer treatment, which augmented her loneliness and depression [46]. Some patients found that cancer was regarded as more socially acceptable than mental illness, and it was easier to obtain support and help for problems and concerns related to cancer than SMI [44].

### 3.3. Decision-Making Capacity Depending on the Assessor

The patients were met with different assessments regarding their decision-making capacity in the fields of psychiatry and cancer care, respectively, which could negatively influence their treatment and support possibilities within cancer care services [42,43]. Kotze and Ross [42] described assessments of the decision-making capacity about end-of-life care in a woman with schizophrenia and cancer. The patient’s assessment, measured through an assessment scale the healthcare professional handed her, showed that she valued quality-of-life more highly than life-length in cases of severe illness, in which case she would decline life-prolonging interventions. Contrary to these preferences, she wanted cardiopulmonary resuscitation if facing a heart attack or a debilitating stroke. In contrast to the psychiatrist, the cancer care physicians concluded that she did not have end-of-life decision-making capacity [42]. Lyckholm and Aburizik [43] presented an example of a man with schizophrenia and cancer. During the initial surgical consultation, he did not believe that he had cancer and refused treatment. Later, he acknowledged the diagnosis and considered surgery. However, the surgical cancer care team opted against surgical intervention considering that the patient did not demonstrate a reliable understanding of the diagnosis, representing a higher risk of postoperative complications. The surgeons deemed it unsafe to operate but offered no further explanation to the patient [43]. The intervention of other clinicians such as psychiatrists or significant others could mean a divergent assessment and revised treatment decisions for the patients, affecting their life expectancy and prospects [41,42,43,44].

Kotza and Ross [42] suggested a pragmatic approach focused on the practical outcomes of assessments of patients’ decision-making capacity by assessing whether the patient’s capacity actually changes treatment choices, and to decide from there. Lyckholm and Aburizik [43] further pinpointed the necessity of compassionate, empathetic, and imaginative care, as a person’s decision-making capacity involved multiple components, i.e., comprehending, evaluating, and choosing. This required non-judgmental care by healthcare professionals to properly attend to vulnerable patients with comorbidities, who might not fully comprehend the meaning of their cancer diagnosis, treatment, and prognosis [43].

When patients’ views or abilities to understand were deemed to limit their compliance with the physicians’ suggested treatments, the treating cancer physicians could however label patients as ‘difficult’ and ‘unfit’ candidates for treatment [40,42]. Nonetheless, one study showed positive patient experiences of somatic care, with experiences of empowerment and of not being stigmatised for one’s psychiatric condition [44]. Some studies directly or indirectly broached the subject of attitudes, stigma, and discrimination against persons with SMI [40,42,43,46], recognising these as issues affecting patients’ everyday lives. Paradoxically, one article simultaneously displayed a language that could be interpreted as stigmatising and discriminating, e.g., when writing “the mentally ill” ([40], p. 778) rather than “persons with mental illness”. The article had a tone testifying an understanding of individuals with SMI as troublesome and strange, using an ‘us-them’ labelling [40]. Peterson and Cunningham [44], on the other hand, showed that individuals with SMI were able to partake in decisions and advocate for themselves, challenging the stereotypes of these patients as difficult or unable to cooperate in their own care.

From an ethical perspective, some studies highlighted that patients’ autonomy, free will, and participation in decision-making about treatment or end-of-life care could be infringed, with risks of discrimination and treatment disparities [42,43,45,46]. Cognitive and physical impairments and vulnerabilities, including medical complexity and lack of disease insight, could affect patients’ everyday lives and care decisions. Lack of knowledge and understanding of life with comorbidities across somatic and psychiatric services could likewise become a hindrance for treatment in persons with SMI and cancer, contributing to treatment disparities with effects on life expectancy. Several studies highlighted treatment challenges and suggested interventions to facilitate the care of persons with SMI and cancer comorbidities, taking into consideration the specific vulnerabilities and challenges encountered by both patients and healthcare professionals [40,41,42,43,45,46]. The risk of suicide could be an imminent threat in patients with SMI, not least when affected by an additional, potentially life-threatening cancer diagnosis [41,46]. Such emergencies framed the patients’ participation in decision-making about their care and required hospitalisation and coercive care. Thomson and Henry [46] highlighted that preparedness for psychiatric emergencies, including suicide risk, and adequate interventions must be considered, including education of nurses in cancer care. Education was understood as a bridge to facilitate the identification of mental health issues in patients with comorbidities.

### 3.4. Cancer Must Give Way to Severe Mental Illness or Vice Versa

Three studies indicated that the SMI and cancer diagnoses tended to overrule each other, i.e., the patients’ lives were primarily marked and steered by one of the diagnoses. It seemed difficult to juxtapose the two diagnoses in everyday life [44,45,46]. Additionally, the cancer diagnosis and treatment could lead to decompensation of the psychiatric condition, thereby increasing the patient’s vulnerability [41]. Thomson and Henry’s [46] first example was a man with bipolar disorder and cancer. He stated that he had cancer, and hence, that he did not need medication for his bipolar disorder. The second example was a woman diagnosed with severe schizophrenia and advanced cancer. She started treatment with chemotherapy but discontinued the cancer treatment due to exhaustion, which the physicians attributed to the patient’s SMI, and was instead enrolled in end-of-life care [46]. Taylor and colleagues’ [45] case was a homeless woman who was brought to the emergency room by law enforcement officials. The patient was suspected to have widespread cancer, which required a biopsy. She refused treatment. She was transferred to a psychiatric clinic for further stabilisation, which she left against medical advice before she could be transferred back for biopsy [45]. This illustrated how SMI served as an overarching understanding of cancer diseases, which seemed less important for the patients’ understanding of life than their well-known, although often painful, everyday lives with SMI. It meant that patients’ wills and potential disease insights could prevent adequate treatment. This could be seen if only one disease at a time, cancer or SMI, could be at the forefront of the individuals’ everyday lives.

Peterson and Cunningham [44] showed that some women were surprised at how good their mental state was throughout the cancer treatment’s course. However, they expressed a fear of relapse in their SMI after ending oncological treatment [44]. Peterson and Cunningham [44] described that some women sought active support in relation to their mental health situation after the cancer treatment course, either from established contacts within mental healthcare services or through cancer care services. In other words, when the cancer disease was under control, there was room again to consider mental illness in everyday life.

Additionally, the patients’ experiences of SMI could be contrasted with problems in everyday life related to cancer and cancer treatment. The interviewed women described that cancer had less impact on their lives than mental illness [44]. The study also highlighted situations where experiences from women’s lives with SMI transformed cancer diagnoses and treatments into ‘a piece of cake’. For instance, a woman who no longer felt suicidal after cancer diagnosis expressed that the worst thing that would happen was that she could die of cancer [44].

### 3.5. Significant Others as a Safety Net

Families or significant others were important for individuals with SMI, also in relation to their cancer diagnosis [41,42,43,44]. Studies pinpointed the risks associated with a lack of social support when dealing with severe illness and precarious living conditions. As relationships may be difficult and social support lacking in persons with SMI, social workers, clinicians, and volunteers could represent significant sources of social support that can help patients cope with challenging life situations [40]. Kotze and Roos [42] exemplified this with a woman who identified her self-care ability, relationships with family and friends, and a life without significant pain or discomfort as the most essential elements in life. She acknowledged her family but did not want it to be involved in treatment decisions [42]. Conversely, Guan et al. [41] showed how a patient’s sister was heavily involved in care-related consent processes as she possessed a power of attorney for the patient. She was thus active in making decisions to move forward with examinations and treatments/surgery with the patient’s consent. However, from the physicians’ perspective, the patient had difficulties following treatment-related instructions and routines [41]. Furthermore, Peterson and Cunningham [44] showed how significant others functioned as advocates and mouthpieces for individuals with SMI and cancer comorbidities in the cancer care field; for instance, for a woman experiencing that the oncologist did not understand the importance of proceeding with her mental health treatment. The patient’s partner advocated with the oncologist on her behalf, arguing for concurrent oncological and psychiatric treatment [44]. Lyckholm and Aburizik [43] showed how family members functioned as bridge builders between professionals in cancer care services and patients with comorbidities, exemplified by the sister of a man with schizophrenia and cancer. She had a healthcare power of attorney and assisted her brother in making decisions regarding his health, explaining clinical information to him in an understandable manner [43].

## 4. Discussion

This discussion focuses on three main findings. First, we discuss how the everyday lives of patients with SMI and cancer comorbidities are influenced by a body–mind dichotomy perspective within the healthcare profession and in somatic and psychiatric care. Second, we discuss challenges in focusing on patients with SMI and cancer comorbidities within a diagnosis-specialised healthcare system, where diagnoses control the medical gaze more than a single individual’s overall situation. Third, we discuss the fact that professionals apprehended patients with SMI and cancer comorbidities as challenging, non-compliant, and less capable patients. Finally, we discuss the current study’s strengths and limitations.

Different logics of care, organisation, and specialisation of medicine can be at play in what can be interpreted as a dichotomisation of body–mind. The included studies displayed a body–mind dichotomy, with a clear separation of somatic and psychiatric knowledge fields and care provision. Western medicine is marked by the Cartesian dualism of body–mind, with consequences for care organisation and care possibilities [47]. Most studies were written from the perspective of cancer care professionals, although several studies pinpointed the need for interdisciplinary care and knowledge enhancement across disciplines. In some cases, patients or their significant others acted as a bridge between somatic and psychiatric care. In other cases, healthcare professionals had a willingness to act as a bridge between the different care providers. Integrated care has been put forward as a way to better address the complex needs of patients with concurrent SMI and cancer [48,49]. The findings show potential tendencies of cancer care professionals to treat ‘common life problems’, including existential problems such as loneliness, as pathological conditions. This calls for an awareness of medicalisation and pathologisation of everyday life [50,51].

Through the current review’s systematic literature search, it became apparent that there were numerous articles dealing with the screening and treatment of mental disorders or symptoms in cancer patients, developed in connection with cancer diagnoses and treatments. A cancer diagnosis can represent a psychological trauma, possibly causing post-traumatic stress disorder, anxiety, and depression [52,53]. This is in stark contrast to the current, barely explored research interest, which investigates the opposite movement: being diagnosed with SMI, then developing cancer. Studies show SMI-associated disparities regarding cancer care [19,54,55], including access to psychosocial support [56]. This may be reflected in the hierarchy of diseases in the medical field, where cancer diseases are regarded as high-prestige diagnoses and psychiatric disorders as low-prestige diagnoses [57,58]. Diseases and specialties dealing with chronic conditions that lack clear-cut bodily location and with less noticeable treatment procedures have lower prestige than diseases associated with technologically sophisticated, urgent, and invasive procedures in vital organs. The specialisation-specific starting point in medical care seems to represent a challenge in the fields of both cancer and psychiatry in regard to acknowledging the everyday life and associated difficulties of individuals with SMI and cancer comorbidities. In the cancer field, cancer-related psychiatric disorders/symptoms are prominent topics within their own ‘psychosocial oncology’ or ‘psycho-oncology’ subfield [59,60]. In the psychiatric field, the everyday lives of patients with concomitant SMI and cancer seems to be an overlooked research area. However, there is a focus on managing physical health issues in everyday life in psychiatric settings, aiming to act on health disparities [61].

Patients’ encounters with healthcare professionals might influence their everyday lives and the treatment of SMI and cancer. As seen in the current findings, healthcare professionals could view patients with SMI as ‘unfit’ for cancer treatment/care, which was due to the professionals’ apprehension of the patients’ SMI. Being viewed as a challenging, noncompliant, and less capable patient might also affect a person’s sense of self-efficacy, control, and agency. Ethical and care principles, such as striving for autonomy, empowerment, voluntary care, and care equity can also be jeopardised. This may be especially true in cases with little knowledge about SMI and comorbidities, and the occurrence of stigma and discrimination. The current review’s case studies showed that patients cared about quality-of-life, sometimes to the detriment of life years, regardless of disease insight and experiences in encounters with healthcare professionals. Distinguishing between lack of insight and will is hence essential in care decisions, whether affected by SMI or not [62]. In line with this, Nordentoft and colleagues [22] show that individuals with SMI have the same interest as the population in general in addressing smoking, diet, and physical activity-related lifestyles, leading to enhanced health and quality-of-life and meaningful everyday lives [22]. In the current study, physicians’ voices resound, the voices of individuals with SMI are nearly absent, and significant others’ voices are absent. There is a clear knowledge and research gap related to the representation of patients’ and significant others’ voices from their own perspective, calling for further studies giving voice to the voiceless. Potential explanations may include stereotypes, misdirected intentions to protect vulnerable patients, and an underestimation by healthcare professionals and researchers of the capacity of persons with SMI to assess their health, voice their needs, and participate in research [63]. Studies show that persons with SMI are capable of self-assessment and participation in research, an approach that goes in line with the strive towards co-production and user involvement in care and research, as seen in Western countries [64,65,66,67].

The current review has strengths and limitations. The construction of search strings resulted in a high number of hits. The construction was discussed with a university librarian, and it was not possible to limit it further. There were several causes for these limitation problems. All desired articles were to deal with people who had both SMI and cancer. When the keywords, for example, contained ‘cancer AND depression’, all cancer-related depressions also appeared, which was not the current study’s focus. Another example consisted of the search words [cancer AND bipolar], which resulted in studies about bipolar psychiatric diagnosis, bipolar radiofrequency ablation, bipolar androgen therapy, etc. Another problem was that studies about people with cancer that had excluded individuals with ‘psychiatric disorders’ also appeared in the search. Additionally, the Boolean operator ‘NOT’ was not used to reduce the number of hits as the risk of sorting out relevant studies was high. This was the reason for not inserting a bar for, for example, randomised controlled studies, quantitative studies, and children. Therefore, an extensive manual screening was needed to find the relevant articles. Additional keywords related to the patients, such as ‘survivor’ or ‘citizen’, and keywords encompassing additional healthcare professions, such as ‘social worker’ and ‘nurse assistant’, may generate additional hits, thereby potentially affecting the results. The studies fulfilled most of the applicable assessment criteria of case studies and qualitative interview studies, respectively, except for two more lowly-ranked case studies. The studies represented a professional, clinical healthcare perspective, with one exception [44]. The paucity of research in this area is supported by the fact that Millman and colleagues’ [4] study protocols about the experience of cancer and cancer services in people with SMI never resulted in publications. Leahy et al. [11] pinpoint that their qualitative review, including six studies, is the first of its kind to identify key barriers and facilitators to accessing cancer care for individuals with significant mental health difficulties. The current review includes studies with low evidence pertaining to the studies’ case format, representing a limitation. Nonetheless, it includes all found studies of relevance and points to a suboptimally researched healthcare area. Further interpretations and synthesising of the primary studies to generate new interpretive constructs, explanations, or hypotheses [35,68,69] are always possible and may be developed further as additional studies are being published. The current review sheds light on several challenges encountered by persons with cancer and SMI that clearly prompt further studies to better address their needs for healthcare and support in everyday life. Thematic synthesis is acknowledged as a useful interpretive approach to synthesise and summarise existing knowledge, but if the aim is to provide recommendations for practice, meta-aggregation is, according to JBI, the preferred approach [70]. A meta-aggregation would have required qualitative studies with higher quality than is currently available within the studied field.

## 5. Conclusions

The current qualitative systematic review showed that research focusing on experiences of everyday life among individuals with concomitant SMI and cancer was sparse, implying that the current findings should be interpreted with caution. This is per se a significant finding, as it shows that more research focusing on the lived experiences of the targeted population is called for. Furthermore, the findings show that individuals with concomitant SMI and cancer face multiple challenges in their everyday life, where the healthcare system’s organisation and healthcare professionals’ assessments and attitudes can play an important part. There were five case studies from the perspective of healthcare professionals, all from cancer care services. The patients’ perspectives were found in one interview study only, and significant others’ voices were absent. The everyday lives of patients with SMI and cancer comorbidities were influenced by a body–mind dichotomy perspective within the healthcare profession and between somatic and psychiatric care. Patients were met with different assessments regarding their decision-making capacity in the fields of psychiatry and cancer care, respectively. Patients were at times regarded as challenging, non-compliant, unfit for treatment, and less capable patients in cancer care services, which might influence their everyday lives and treatments. SMI and cancer diagnoses tended to overrule each other, meaning that the patients’ everyday lives were primarily steered by one of the diagnoses. It was difficult for both patients and healthcare professionals to act simultaneously in two different fields of medicine. However, significant others functioned as bridge builders between the two fields. The study calls for further research exploring the complexity of everyday life among individuals with SMI and cancer comorbidities, including patients’ and significant others’ perspectives. Furthermore, we suggest anthropological field studies exploring the (inter)actions in the psychiatric and cancer care fields between healthcare professionals, individuals with SMI and cancer, and their significant others.

## Figures and Tables

**Table 1:** Populations, exposures, and outcomes (PEO) customised to each database.

Block 1 (P)—Population	Block 2 (E)—Exposure	Block 3 (O)—Outcome/Theme
Patients, healthcare professionals, relatives	Cancer and severe mental illness	Experiences
Patient* OR Client* OR Citizen* OR Physician* OR Doctor* OR Nurse* OR Health care professional* OR Health care worker* OR Healthcare professional* OR Healthcare worker* OR Nursing staff OR Medical staff OR Community Health Workers OR Relative* OR Famil* OR Next of kin* OR Spouse* OR Informal carer* OR Caregiver* OR Sibling* OR Significant other* OR Significant other OR Extended family*	Cancer OR Malignant disease* OR Malignancy OR Neoplasms OR malign*Carcinoma* OR Lymphoma* OR Melanoma* OR Neuroblastoma* OR Neoplasms* AND Psychiatric disorder* OR Psychiatric disease* OR Severe mental Illness OR Severe mental disease* OR Schizophrenia* OR Bipolar disorder* OR Non-organic psychoses OR Major depressive disorder* OR Mental Disorders OR Schizophrenia, Disorganized OR Schizotypal Personality Disorder OR Schizophrenia, Catatonic OR Schizophrenia, Paranoid OR Schizophrenia Spectrum and Other Psychotic Disorders OR Depressive Disorder OR Bipolar Disorder OR Depressive Disorder, Major OR Borderline Personality Disorder OR mental disord* OR psychiatric disorder* OR bipolar* OR psychosis OR affective disorder*	Experience* OR Everyday Life OR Encount* OR Involvement OR Daily living OR Participation OR Shared decision making OR Relationship* OR Interaction* OR Communication OR Information OR Activities of Daily Living OR Interpersonal Relations OR Communication OR Stakeholder OR Participation OR Social Participation OR Patient Participation OR Community Participation OR Adaptation, Psychological OR Information Dissemination OR Consumer Health Information OR life experience* OR activities of daily living OR participat* OR involv* OR decision making OR interpersonal relation* OR communicat* OR informat* OR coping* OR cope*

Examples of search terms from the PubMed search (for the full search strings see Appendix A).

**Table 2:** Information extracted from the articles.

Author(s), Year of Publication; Study Location	Journal (Year: Impact Factor)	Study Aim	Design; Study Population	Study Period
Cole & Padmanabhan, 2012; USA [40]	Journal of Cancer Education (2019: 1.576)	To illustrate the obstacles women with concurrent severe mental illness face in maintaining adequate cancer care.	Case study; Three patients	Unknown
Guan et al., 2021; USA [41]	Cureus (2020–2021: 1.15)	To explore the possibility of careful communication between the treating physician, patient, and the patient’s caretakers potentially preventing the delay in a patient’s cancer diagnosis.	Case study; One patient	Unknown
Kotze & Roos, 2020; South Africa [42]	South African Family Practice (2019: N/A)	To address some of the difficulties with discrepancies between clinical and formal capacity assessments in end-of-life decision-making capacity in a female with treatment-resistant schizophrenia and terminal cancer	Case study; One patient	2018–2019
Lyckholm & Aburizik, 2017; USA [43]	AMA Journal of Ethics (2018: 0.98)	To explore practical and ethical issues surrounding the care of patients with mental illness and cancer.	Case study; One patient	Unknown
Peterson & Cunningham, 2020: New Zealand [44]	Kōtuitui: New Zealand Journal of Social Sciences Online (2019: N/A)	To explore the experiences of women with mental illness who had also experienced breast cancer diagnosis and treatment; in particular, to identify barriers and facilitators to cancer treatment.	Semi-structured interviews; Ten patients	2016
Taylor, Golas, Martel, & Martel, 2013; USA [45]	Ophthalmic Plastic and Reconstructive Surgery (2019: 1.331)	To illuminate the complexities of managing a patient with a schizoaffective disorder and advanced basal cell carcinoma	Case study; One patient	2009–2011
Thomson & Henry, 2012; USA [46]	Clinical Journal of Oncology Nursing (2019: 1.224)	To examine problems that patients with severe mental disorders encounter with their cancer diagnosis and treatment.	Case study; Four patients	Unknown

## Data Availability

All included articles are publicly available.

## Appendix A

**Table A1:** The full electronic search string for CINAHL, PsychInfo, and PubMed/MEDLINE.

Database	Search String
**CINAHL** Search date: 15 June 2021 and 14 February 2023 Limiters: Published Date: 1 January 2011–14 February 2023 English Language Research Article Hit 4791 + 995	(((((MM “Patients+”)) OR (MH “Cancer Patients”) OR “client*” OR “citizen*” OR ((MH “Physicians+”)) OR “doctor*” OR ((MH”Nurses+”)) OR “nurses” OR “nurse” OR (“health care professional”) OR (“healthcare professional”) OR (“healthcare worker”) OR (“relative*” OR (MM” Extended Family+”)) OR ((MH “Family+”) OR “famil*”) OR (“next of kin”) OR (“spouse*” OR (MH “Caregivers”) OR (MH “Significant Other”) OR (MH “Spouses”)) OR ((MH “Siblings”) OR “sibling*”) OR ((MH “Significant Other”) OR “significant other*”) OR (“informal carer*”) OR “caregiver*”)) OR (AB (patient* OR client* OR citizen*) OR TI (patient* OR client* OR citizen*) (MH “Physicians+”) OR (MH “Medical Staff+”) OR (MH “Nursing Manpower+”) AB (doctor* OR nurse* OR “health care professional*” OR “healthcare professional* OR “healthcare worker*”) OR TI (doctor* OR nurse* OR “health care professional*” OR “healthcare professional* OR “healthcare worker*”)(MH “Extended Family+”) TI (relatives OR famil*OR spouse* OR caregiver* OR sibling* OR carer) AND AB relatives OR famil* OR spouse* OR caregiver* OR sibling* OR carer) OR ((MH “Family+”) OR (MH “Significant Other”) OR (MH “Significant Other”) OR (MH “Spouses”) OR (MH “Siblings”)) OR (Relative* OR Famil* OR Next of kin* OR Spouse* OR Informal carer* OR Caregiver* OR Sibling* OR Significant other*) OR (““Physician* OR Doctor* OR Nurse* OR Health care professional* OR Health care worker* OR Healthcare professional* OR Healthcare worker*”“) OR (““Patient* OR Client* OR Citizen*”“)) AND (((TX (Cancer OR Malignant disease* OR Carcinoma OR Lymphoma OR Malignancy OR Melanoma OR Neuroblastoma)) OR ((MH “Neoplasms+”)) OR (TI (cancer OR malign*) OR AB (cancer OR malign*)) OR (“cancer” OR (“malignant disease”) OR ((MH “Carcinoma+”) OR “carcinoma”) OR ((MH “Lymphoma+”) OR “lymphoma”) OR “malignancy” OR “Melanoma”)) AND ((TX (Psychiatric disorder* OR Psychiatric disease* OR Severe mental Illness OR Severe mental disease* OR Schizophrenia OR Bipolar disorder* OR Non-organic psychoses OR Major depressive disorder*)) OR ((MH “Mental Disorders+”)) OR (TI (“psychiatric disease*” OR “psychiatric disorder*” OR “severe mental illness*” OR “severe mental disorder*” OR SMI OR “severe psychiatric illness*” OR SPI OR schizophrenia OR bipolar OR psychosis OR psychoses OR “affective disorder*” OR “major depressive disorder*”) OR AB (“psychiatric disease*” OR “psychiatric disorder*” OR “severe mental illness*” OR “severe mental disorder*” OR SMI OR “severe psychiatric illness*” OR SPI OR schizophrenia OR bipolar OR psychosis OR psychoses OR “affective disorder*” OR “major depressive disorder*”)) OR (((“Psychiatric disorder”) OR (“Psychiatric disease”) OR ((MH “Mental Disorders+”)) OR (“Severe mental Illness” OR SMI OR (MM “Mental Disorders, Chronic”) OR (MM “Mental Disorders+”)) OR ((MM “Mental Disorders, Chronic”) OR “Severe mental disease”) OR ((MM “Schizophrenia+”) OR “Schizophrenia”) OR ((MM “Schizophrenia+”) OR “Schizophrenia”) OR ((MM “Bipolar Disorder+”) OR “Bipolar disorder”) OR (“Non-organic psychoses”) OR ((MH “Bipolar Disorder+”) OR “Major depressive disorder” OR (MM “Affective Disorders, Psychotic+”)))))) AND ((((MM “Life Experiences+”) OR “Experienc*”) OR (“everyday life”) OR (“daily living” OR (MM “Activities of Daily Living+”)) OR “Encounter*” OR MH “ OR (MM “Family OR (MH “Social Participation”) OR (MM “Consumer Participation”) OR (MM “Stakeholder Participation”) OR “involv*” OR MM “ OR “participat*” OR ((MM “Decision Making, Shared”) OR (MM “Decision Making, Family”) OR (MH “Decision Making, Clinical+”) OR (MM “Decision Making+”)) OR ((MH “ OR (MM “Social OR (MM “Interpersonal Relations+”) OR “interaction*”) OR ((MM “Communication+”) OR “Communication”) OR “Information”) OR (MH “Coping+) OR involv* OR inform*(?) OR engag*) OR ((MH “Life Experiences+”) OR (MH “Activities of Daily Living+”) OR “Experience* OR Everyday Life OR Encounter* OR Involvement OR Daily living OR Participation OR Shared decision making OR Relationship* OR Interaction* OR Communica* OR Informa*”) OR ((MH “Activities of Daily Living+”) OR (MH “Life Experiences+”) OR “(participat* OR involv* OR inform* OR engag*) OR engag* OR everyday life OR involv* OR (coping OR cope) OR daily living OR experienc* OR encounter*”) OR (AB coping OR cope OR “everyday life” OR “daily living” OR experienc* OR encounter* OR participat* OR involv* OR inform* OR engag*) OR (TI coping OR cope OR “everyday life” OR “daily living” OR experienc* OR encounter* OR participat* OR involv* OR inform* OR engag*))))
**PsychInfo** Search date 15 June 2021 + 14 February 2023 Limiters: Peer Reviewed Journal Published: 1 January 2011–14 February 2023 Hit: 991 + 177	https://ovidsp.ovid.com/ovidweb.cgi?T=JS&NEWS=N&PAGE=main&SHAREDSEARCHID=6yUSce1XLH0w3qgp5mMz11DqTYhkLmLyhCiBYx74MMROohqLPcnBiazyOPnwC8H2D (accessed on 5 June 2023).

	Query
	exp Terminally Ill Patients/or exp Patients/or patient.mp. or exp Medical Patients/or exp Psychiatric Patients/or exp Hospitalized Patients/or exp Geriatric Patients/or exp Surgical Patients/
	exp Physicians/or exp Family Physicians/or physicians.mp.
	exp Physicians/or exp Medical Personnel/or exp Health Personnel/or medical staff.mp. or exp Nurses/
	health care professional.mp.
	healthcare professional.mp.
	citizen.mp.
	client.mp. or exp Clients/
	family.mp. or exp Family/
	exp Caregivers/or relative.mp.
	relativ*.mp.
	exp Caregivers/or caregive*.mp.
	spouses.mp. or exp Spouses/
	sibling.mp. or exp Siblings/
	significant other.mp. or exp Significant Others/
	informal caregiver.mp.
	1 or 2 or 3 or 4 or 5 or 6 or 7 or 8 or 9 or 10 or 11 or 12 or 13 or 14 or 15
	cancer.mp. or exp Neoplasms/
	Malignant disease*.mp.
	Carcinoma.mp.
	Lymphoma.mp.
	Melanoma.mp. or exp Melanoma/
	exp “Blood and Lymphatic Disorders”/or Malignancy.mp.
	malignant disease.mp.
	Neuroblastoma.mp.
	17 or 18 or 19 or 20 or 21 or 22 or 23 or 24
	psychiatric disorder.mp. or exp Mental Disorders/
	exp Schizophrenia/or Psychiatric disease*.mp.
	exp Chronic Mental Illness/or exp Serious Mental Illness/or Severe mental Illnes*.mp.
	exp Psychosis/or exp Schizophrenia/or Schizophreni*.mp.
	exp Bipolar Disorder/or exp Major Depression/or Bipolar disorder*.mp.
	Major depressive disorder*.mp.
	psychosi*.mp. or exp Affective Psychosis/or exp Acute Psychosis/or exp Psychosis/or exp Reactive Psychosis/
	affective disorder.mp. or exp Affective Disorders/
	26 or 27 or 28 or 29 or 30 or 31 or 32 or 33
	25 and 34
	life experienc*.mp. or exp Life Experiences/
	experienc*.mp.
	exp Daily Activities/or exp “Activities of Daily Living”/or everyday life.mp.
	encount*.mp.
	exp Client Participation/or exp Participation/or participation.mp.
	invol*.mp.
	exp Decision Making/or exp Cooperation/or shared decision making.mp.
	relation*.mp.
	exp Information/or information*.mp. or exp Health Information/
	coping*.mp. or exp Coping Behavior/
	cope*.mp.
	engag*.mp.
	36 or 37 or 38 or 39 or 40 or 41 or 42 or 43 or 44 or 45 or 46 or 47
	16 and 35 and 48
	49 and “Peer Reviewed Journal”.sa_pubt.
**PubMed, primary searching in MEDLINE** Search date: 15 June 2021 Filters: Published date: 1 January 2011–14 February 2023 English Hits: 3114 + 781	Search: (((((((((((patients[MeSH Major Topic]) OR (nurses[MeSH Major Topic])) OR (Nursing Staff[MeSH Major Topic])) OR (Medical Staff[MeSH Major Topic])) OR (Nursing Staff, Hospital[MeSH Major Topic])) OR (Medical Staff, Hospital[MeSH Major Topic])) OR (Physicians[MeSH Major Topic])) OR (Health Personnel[MeSH Major Topic])) OR (Community Health Workers[MeSH Major Topic])) OR (Family[MeSH Major Topic])) OR (((((((((((((patient*[Title/Abstract]) OR (client*[Title/Abstract])) OR (citizen*[Title/Abstract])) OR (Physician*[Title/Abstract] OR Doctor*[Title/Abstract] OR Nurse*[Title/Abstract] OR Health care professional*[Title/Abstract] OR Health care worker*[Title/Abstract] OR Healthcare professional*[Title/Abstract] OR Healthcare worker*[Title/Abstract])) OR (caregiv*[Title/Abstract])) OR (significant other*[Title/Abstract])) OR (spouse*[Title/Abstract])) OR (sibling*[Title/Abstract])) OR (informal carer*[Title/Abstract])) OR (relative*[Title/Abstract])) OR (extended family*[Title/Abstract])) OR (famil*[Title/Abstract])) OR (Health care professional*[Title/Abstract]))) AND( ((((((((((((Mental Disorders[MeSH Major Topic]) OR (Schizophrenia[MeSH Major Topic])) OR (Schizophrenia, Disorganized[MeSH Major Topic])) OR (Schizotypal Personality Disorder[MeSH Major Topic])) OR (Schizophrenia, Catatonic[MeSH Major Topic])) OR (Schizophrenia, Paranoid[MeSH Major Topic])) OR (Schizophrenia Spectrum and Other Psychotic Disorders[MeSH Major Topic])) OR (Depressive Disorder[MeSH Major Topic])) OR (Bipolar Disorder[MeSH Major Topic])) OR (Depressive Disorder, Major[MeSH Major Topic])) OR (Borderline Personality Disorder[MeSH Major Topic])) OR ((((((((((((((((mental disord*[Title/Abstract]) OR (Severe mental disease*[Title/Abstract])) OR (Schizophr*[Title/Abstract])) OR (Bipolar disorder*[Title/Abstract])) OR (Non-organic psychos*[Title/Abstract])) OR (Major depressive disorder*[Title/Abstract]))) OR (psychiatric disease*[Title/Abstract])) OR (psychiatric disorder*[Title/Abstract])) OR (severe mental illness*[Title/Abstract])) OR (severe mental disord*[Title/Abstract])) OR (schizophrenia*[Title/Abstract])) OR (bipolar*[Title/Abstract])) OR (psychosis[Title/Abstract])) OR (psychoses[Title/Abstract])) OR (affective disorder*[Title/Abstract]))) AND ((Neoplasms[MeSH Major Topic]) OR (((((((malign*[Title/Abstract])) OR (cancer*[Title/Abstract])) OR (Carcinoma*[Title/Abstract])) OR (Lymphoma*[Title/Abstract])) OR (Melanoma*[Title/Abstract]) OR (Neuroblastoma*[Title/Abstract])) OR (Neoplasms*[Title/Abstract])))) AND(((((((((((Activities of Daily Living[MeSH Major Topic]) OR (Interpersonal Relations[MeSH Major Topic])) OR (Communication[MeSH Major Topic])) OR (Stakeholder Participation[MeSH Major Topic])) OR (Social Participation[MeSH Major Topic])) OR (Patient Participation[MeSH Major Topic])) OR (Community Participation[MeSH Major Topic])) OR (Adaptation, Psychological[MeSH Major Topic])) OR (Information Dissemination[MeSH Major Topic])) OR (Consumer Health Information[MeSH Major Topic])) OR (((((((((((((((life experience[Title/Abstract]) OR (experienc*[Title/Abstract])) OR (everyday life[Title/Abstract])) OR (daily living[Title/Abstract])) OR (activities of daily living[Title/Abstract])) OR (encount*[Title/Abstract])) OR (participat*[Title/Abstract])) ) OR (involv*[Title/Abstract])) OR (decision making[Title/Abstract])) OR (interpersonal relation*[Title/Abstract])) OR (communicat*[Title/Abstract])) OR (informat*[Title/Abstract])) OR (coping*[Title/Abstract])) OR (cope*[Title/Abstract])))
